# Electrochemistry Test Strip as Platform for In Situ Detection of Blood Levels of Antipsychotic Clozapine in Finger-Pricked Sample Volume

**DOI:** 10.3390/bios13030346

**Published:** 2023-03-04

**Authors:** Mehmet Senel

**Affiliations:** Department of Biochemistry, Faculty of Pharmacy, Biruni University, Istanbul 34010, Turkey; msenel@biruni.edu.tr

**Keywords:** electrochemical sensor, clozapine, test strips, point of care testing

## Abstract

With the increasing number of patients suffering from Parkinson’s disease, the importance of measuring drug levels in patient body fluids has increased exponentially, particularly for the drug clozapine. There is a growing demand for real-time analysis of biofluids on a single low-cost platform in ultra-low fluid volumes with robustness. This study aims to measure the level of clozapine (Clz) with a portable potentiostat using a practical approach. For this purpose, we developed an inexpensive, portable platform via electrochemistry on a commercial glucose test strip (CTS). CTSs were first modified by removing the enzyme mixture from the surface of the sensing zone, which was followed by modification with Multi walled carbon nanotube (MWCNT) and Nafion. The electrochemical characteristics of CTS electrodes were investigated using cyclic voltammetry (CV) and differential voltammetry (DPV) techniques. The designed sensor displayed decent linear range, detection limit, reproducibility, and reusability results. A linear dynamic range of 0.1–5 μM clozapine was observed under optimized conditions with a good sensitivity (1.295 μA/μM) and detection limit (83 nM). Furthermore, the designed sensing electrode was used to measure the amount of Clz in real samples.

## 1. Introduction

The individualization of therapeutic drug dosing is an emerging field, and the development of a novel method for drug monitoring is crucial in this area. Clozapine (Clz) is a widely used and effective antipsychotic medication that is prescribed to treat patients with schizophrenia. However, determining the appropriate dosage can be difficult due to the drug’s dose-dependent effects [1]. Measuring the levels of Clz in biological and pharmaceutical samples is a crucial step in determining the appropriate dosage, but this can be a barrier to the broader use of clozapine [2]. Several analytical techniques have been developed to determine Clz levels, including capillary zone electrophoresis [3], high-performance liquid chromatography [4], colorimetry [5], capillary gas chromatography [6], and spectrophotometry [7]. However, most of these techniques are complex and require expensive equipment and reagents. Electrochemical analysis, on the other hand, is a simpler and more cost-effective alternative. It offers many advantages over traditional techniques, including low cost, quick response time, simplicity, robustness, and high sensitivity towards low analyte concentrations [8,9,10]. Different types of electrode platforms have been used in electrochemical sensors, including gold electrodes [11], Indium thin oxide (ITO) coated-glass electrodes [12], glassy carbon electrodes [13], and carbon fiber electrodes [14]. Recently, there has been a growing interest in using screen-printed electrodes (SPEs) for electroanalytical research. SPEs offer many benefits, such as easy setup, system miniaturization, and portability, compared to traditional three-electrode systems [15,16].

SPEs have several benefits that make them appealing for various applications. They are easy to mass produce, have a high level of reproducibility, and are relatively inexpensive [17]. Additionally, SPEs have the advantage of being versatile, providing quick response times, and allowing miniaturization. These attributes have led to the development of disposable sensors that can be used to directly analyze drug samples without modification. For example, a previous study demonstrated the ability to detect clozapine (Clz) in both biological and pharmaceutical samples using differential pulse voltammetry with SPEs [18].

Screen-printing technology is widely used for the mass production of disposable glucose test strips. They have been manufactured by coating or deposition of a metallic layer on plastic or paper [19]. On Call is one of these technologies, and it is characterized by small physical dimensions, low cost, high accuracy, and short measurement time for blood glucose estimation. This strip is manufactured by screen printing silver and carbon layers on a plastic substrate.

Recent advancements in electrochemical sensors have led to the use of various modifying agents on the surface of electrodes to improve their sensitivity and selectivity [20,21]. One popular method is the use of nanomaterials, such as carbon-based materials [22], noble metal nanoparticles [23], magnetic nanoparticles [24], and transition metal oxide nanomaterials [25]. These materials help to reduce the overpotentials associated with redox reactions, thus increasing the efficiency of the electrocatalytic process [26,27]. Additionally, previous research has shown that the use of multi-walled carbon nanotubes (MWCNT) can have a positive impact on electrocatalytic activity due to their large electroactive surface area and broad electrochemical window [28,29].

In the present work, the use of washed commercial On Call test strips for instantaneous detection of Clz was described. The performance of the Clz sensor was assessed by voltammetric (CV: Cyclic voltammetry, DPV: Differential pulse voltammetry, and SWV: Square wave voltammetry) and amperometric techniques. The optimized electroanalytical results indicate that the modified commercial test strip (CTS) has a high sensitivity and low Limit of detection (LOD) for the sensing of Clz. Moreover, the sensitivity and selectivity of the modified CTS were excellent towards the analyte, and it was used for Clz detection in very low-volume spiked serum samples with acceptable recovery values.

## 2. Materials and Methods

### 2.1. Materials

All chemicals used in this experiment were of the highest analytical grade and were used without any additional purification. All solutions were prepared using only double-distilled water unless otherwise stated. The chemicals used in this experiment included clozapine, Nafion, MWCNT, uric acid, ascorbic acid, sodium chloride, potassium chloride, potassium hexacyanoferrate (III), potassium hexacyanoferrate (II) trihydrate, sulfuric acid (fuming), hydrochloric acid (37%), and sodium hydroxide, which were all obtained from Sigma Aldrich (Darmstadt, Germany). Real samples of donkey serum were also obtained from Sigma Aldrich (Darmstadt, Germany) for use in the experiment.

### 2.2. Sensor Fabrication

The preparation of the CTS involves three key steps. First, the plastic coating at the end of the electrode was physically removed, and the coating of enzymes and mediators on the original CTS was cleaned off using ethanol and distilled water and then left to air dry. Next, a mixture of MWCNT (in various amounts) and 0.5% Nafion in ethyl alcohol (1 µL) was applied to the tip of the strip substrate and allowed to dry at room temperature.

### 2.3. Electrochemical Measurements

Electrochemical measurements were performed using a Sensit BT portable electrochemical potentiostat (Palmsens, Houten, The Netherlands). The working, counter, and reference electrodes were commercial On Call Plus glucose test strips (ACON, San Diego, CA, USA) and were connected to the potentiostat. All electrochemical measurements were performed at room temperature.

## 3. Results and Discussion

### 3.1. Electrochemical Characteristics

**Figure 1:**
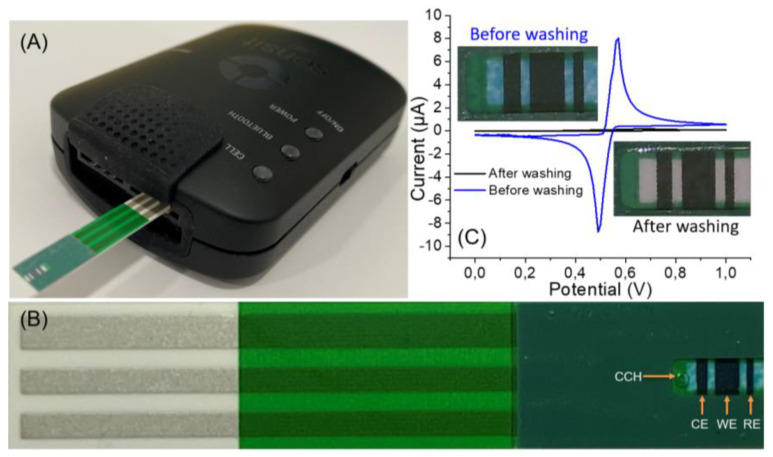
(**A**) Image of CTS inserted into the portable electrochemical reader. (**B**) Image of CTS with the positions of the electrodes. (**C**) CV responses of the CTS and washed CTS in PBS (pH 7.4, 10 mM) with scan rate 100 mV/s. Inset: images of the sensing zone of the unwashed and washed CTS.

**Figure 2:**
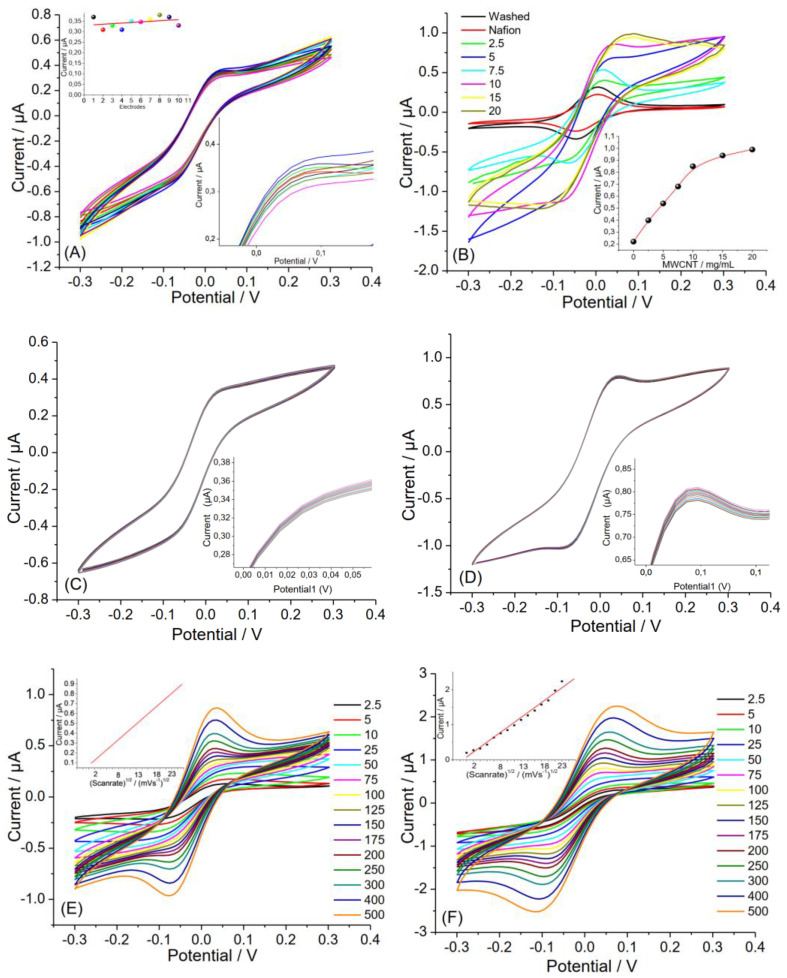
(**A**) CV curves of a randomly selected 10 different washed CTSs (scan rate of 50 mV/s) Inset: number of electrodes vs. sensor response and magnified zone of plot; (**B**) effect of surface modification of CTS with Nafion (Nf) and MWCNT (scan rate of 50 mV/s); repeated CV curves for inset: amount of MWCNT vs. sensor response (**C**) washed and (**D**) Nf/MWNCT-coated CTS (scan rate of 50 mV/s, 20 scans); CV curves of inset: magnified zone of plot (**E**) washed and (**F**) Nf/MWNCT-coated CTS with different scan rates in a solution of 5.0 mM [Fe(CN)_6_]^3−^/^4−^/0.1 M KCl.

Herein, the CTSs were used to develop a Clz sensor for the measurement of Clz levels in serum spiked samples. The representation of the steps followed for the preparation of the sensor is illustrated in Figure 1. First, the CTSs were cleaned with distilled water and ethanol several times to remove all the components of glucose measurement such as the enzyme and mediator. The cleanness of the CTS was evaluated visually by observing the disappearance of the blue color associated with the enzyme (Figure 1C). Additionally, CV was used to evaluate the cleanness of the CTSs. Figure 1C shows the CVs of the washed and un-washed CTSs in phosphate buffer solution PBS (pH 7.0, 10 mM). The intense peak in Figure 1C belongs to the mediator (potassium hexacyanoferrate) on the electrode surface. As seen in the CV of the washed CTS, this intense peak disappeared after the cleaning process. Moreover, the CV of a randomly selected ten electrodes was evaluated in 5 mM potassium ferricyanide in 0.1 M KCl. Figure 2A showed that there was no significant difference between ferrocyanides’ peak intensities with different electrodes. The cleaning process can be used to prepare unique CTSs for biosensor design.

**Figure 3:**
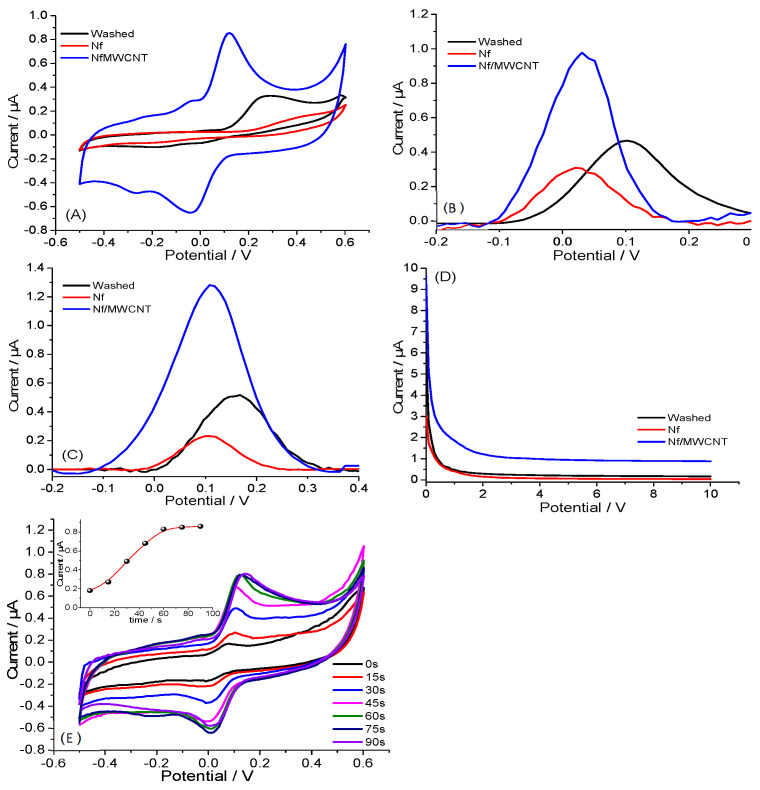
(**A**) CV, (**B**) DPV, (**C**) SWV curves, and (**D**) amperometric response of washed, Nafion (Nf)- and Nf/MWNCT-coated CTSs for 1 µM Clz; (**E**) the influence of accumulation time on Ipa of 1 µM Clz using Nf/MWCNTs in PBS (10 mM, pH 7.4) at a scan rate of 100 mV/s.

After cleaning, the CTS was coated with Nafion solution to create a selective layer that can block interfering substances such as ascorbic acid, uric acid, etc. [11]. The performance of electrochemical sensors increased with the surface modification strategy. Therefore, MWCNT was added to the Nafion solution, while the coating of the CTS surface increased the conductivity of the biosensor [29]. The MWCNT was used with different amounts during CTS electrode modification. It was observed that the sensitivity of the biosensor first increased rapidly with increasing amount of MWCNT in Nafion solution and then reached a plateau at a concentration of 10 mg/mL. Therefore 10 mg/mL of MWNCT was preferred as the optimum concentration and used for further CTS electrode fabrication. Whereas the washed CTS electrode recorded an anodic peak current of 0.32 µA, the Nafion-modified CTS electrode recorded lower currents (0.24 µA), and the Nf/MWCNT-modified CTS electrode recorded higher currents (1 µA) (Figure 3B–D), indicating an increase in the electroactive area of the electrode through electrochemical polarization. Figure 3B,C also show that the washed and Nf/MWCNT-coated CTS electrodes were electrochemically stable after 20 scans. The effect of the scan rate on the cyclic voltammogram of ferrocyanide at the washed CTS and Nf/MWCNT-coated CTS electrodes was investigated. As could be seen in Figure 2E,F, the plots of the anodic peak current (Ip) were linearly dependent on the potential scan rate in a range of 2.5 to 500 mV/s, which shows the adsorption-controlled process under such conditions [28].

Clz is an electro-active, drug-containing diazepine group that has been studied by numerous researchers and can be identified by the electrochemical reaction of the drug [30,31]. Figure 3A–D compares the CV, DPV, SWV curves, and amperometric responses of different electrodes (washed, Nafion-coated, and Nf/MWCNT-coated) in Clz solution (1 µM in 10 mM PBS, pH = 7.4). As expected, the current corresponding to the oxidation at the bare CTS electrode was higher as compared to that of the Nafion-coated CTS electrode, confirming the successful coating of Nafion on the CTS surface. However, such a decrease in peak current is not efficient for sensor development. That is why MWCNT was added to the Nafion solution, and a signal increase was observed during CV, DPV, SWV curves, and amperometric measurements. The accumulation time significantly affected the sensing performance of electrochemical biosensors, especially modified electrodes, due to limitations of diffusion of the analyte to the electrode surface. The effect of accumulation time in the determination of Clz was investigated from the CV curvature of Nf/MWCNT-coated CTS in the presence of 1 µM Clz in 10 mM PBS (pH 7.4) with a scan rate of 100 mV/s as depicted in Figure 3E. The accumulation time was evaluated from 0 to 90 s, and it reached a plateau at 60 s. Therefore, a 60 s accumulation time is suitable for the modified sensor.

### 3.2. Electrochemical Clz Measurements

**Figure 4:**
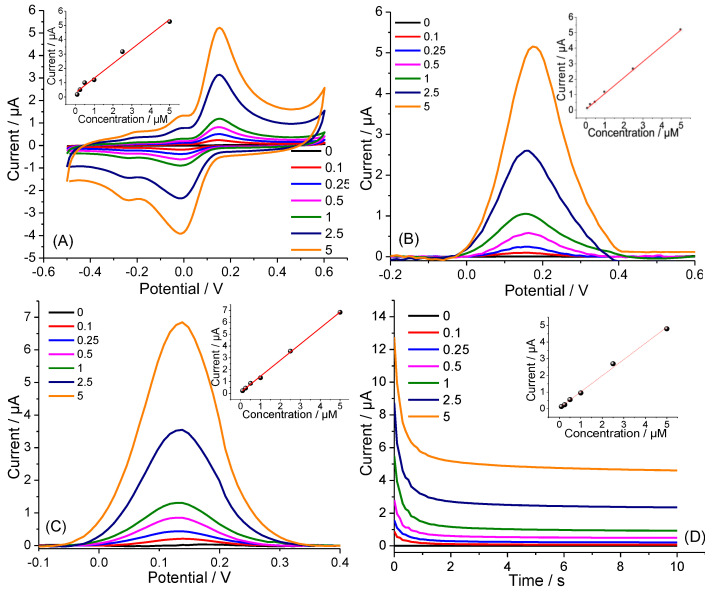
Electroanalytical performance of CTS sensing electrodes toward the detection of Clz in PBS buffer (in 10 mM, pH 7.4) with different concentrations: 0.1, 0.25, 0.5, 1.0, 2.5, and 5 µ Clz. (**A**) CV, (**B**) DPV, (**C**) square-wave voltammogram responses, and (**D**) amperometric responses. Insets show the corresponding calibration plots. Insets: concentration vs. response plots.

The analytical performance of the CTS-based sensing electrodes was tested toward the detection of Clz over the concentration range of 0.1 to 5 μM (in 10 mM PBS, pH 7.4) by using different electroanalytical techniques. The performance of various concentrations of Clz is shown in Figure 4A through voltammograms. There was a strong correlation observed between peak current and Clz concentration in a range from 0.1 to 5 µM, with coefficients of determination near 1. The limit of detection (LD) was calculated using the equation LD = 3 SD/slope, where SD is the standard deviation of background currents, and the slope is from the calibration curve, resulting in an LD of 116 nM. Figure 4B demonstrates the DPV of Clz concentration, with an inset displaying the calibration plot of the reduction peak current against Clz concentrations. As the figure indicates, the peak current increased as the Clz concentration increased. The linear correlation between the oxidation peak current (I, μA) and Clz concentration (C, μM) was found to be strong in the range from 100 nM to 5 μM (R^2^ = 0.9962). The limit of detection (LD) was 104 nM with a signal-to-noise ratio of 3. The sensing performance of the coated CTS towards Clz was also investigated using square-wave voltammetry. As seen in Figure 4C, anodic peaks were centered at 0.15 V, and a linear correlation with Clz concentration was observed between 0.1 and 5 µM (Figure 4C, R^2^ = 0.9962). The detection limit of Clz was 83 nM. Amperometry was also investigated during the electrochemical analysis of Clz because of the possibility to reduce background capacitances and increase the sensitivity of the analysis. Calibration curves were created using amperometric measurements with Clz solutions in the concentration range of 100 nM to 5 μM under the obtained optimized parameters. Figure 4D shows that the Clz oxidation response currents increased with increasing the concentration of Clz. A linear correlation with dopamine concentration was observed between 0.1 and 5 µM (R^2^ = 0.985) as shown in the inset in Figure 4D with a detection limit of (3σ S/N) 192 nM. Consequently, the detection limit was lower using SWV as a detection method.

Table 1 compares the performance of the fabricated sensor to several previously reported methods for measuring Clz. Clozapine has a narrow therapeutic range, and its concentrations in the blood need to be carefully monitored to avoid toxicity or inadequate treatment. The paper’s sensor was able to detect clozapine at clinically relevant concentrations (10–500 ng/mL), which makes it potentially useful for monitoring clozapine levels in patients. The results, particularly those using SWV detection, suggest that the Nf/MWCNT-coated CTS has a wide linear dynamic range and a low DL, which demonstrates the potential of the modified CTS for detecting Clz within the clinically relevant range. In Table 1, there are other electrochemical sensors that have similar or better parameters, such as linear range and limit of detection (LOD), compared to the sensor presented in the paper. Therefore, it is important to highlight the unique features and advantages of the sensor in the paper. This suggests that the fabricated sensor could be a promising candidate for determining trace levels of Clz in real-world samples. Further research will be necessary to enhance the sensor’s sensitivity.

### 3.3. Selectivity, Reproducibility, and Real Sample Analysis

**Figure 5:**
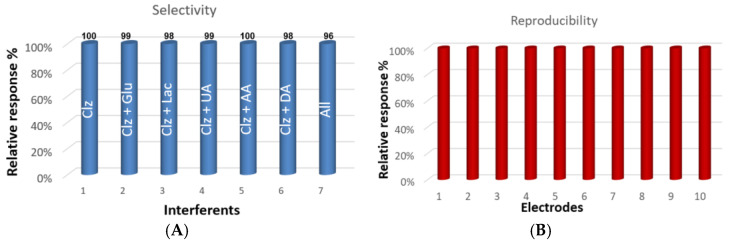
(**A**) Selective CV response of modified CTS towards Clz in the presence of common various interfering species (AA: ascorbic acid, Lac: lactic acid, Glu: glucose, UA: uric acid); (**B**) reproducibility of modified CTS containing 1 µM Clz at ten different electrodes (10 mM PBS, pH 7.4).

In order to analyze biological samples with the proposed modified electrode, the influence of common electrochemical species in blood was investigated using SWV. To assess the sensor’s ability to detect Clz in the presence of coexisting and interfering substances, selectivity studies were conducted using solutions containing Clz (1 μM) and other substances such as glucose (5 mM), uric acid (20 μM), ascorbic acid (20 μM), and lactic acid (5 mM) (Figure 5A). The Nf/MWCNT-coated CTS electrode was able to detect Clz even in the presence of these interferents. The high cationic attraction ability of Nafion and the excellent electron exchange properties of pristine MWCNTs were effectively combined to create a stable sensor with high sensitivity that can eliminate interference from AA and UA at concentrations above their maximum blood levels. This suggests that the developed voltammetric sensor is specific and may be considered a viable alternative for determining and monitoring Clz in biological samples, even in the presence of various interferents.

Furthermore, the reproducibility of the modified electrode was examined by recording SWVs of SWV in multiple measurements with the use of ten Nf/MWCNT-coated CTS electrodes fabricated independently (Figure 5B). The peak currents of the SWVs of 1 μM Clz recorded at these electrodes varied to an extent of only 2.4%. Overall, the results were quite satisfactory and showed that the modified CTS electrode is highly reproducible for accurate electro-catalytic detection of Clz.

We also investigated the applicability of the probe for Clz detection in human serum samples. The recovery test of spiked samples was performed to evaluate the accuracy of the method. SWV responses and the peak currents corresponding to Clz oxidation for different concentrations of samples were collected to perform Clz determination in a spiked serum sample. The measured concentration of Clz was calculated and compared to the actual spiked concentration based on the calibration plot (Figure 4C). The recovery rates were then calculated. As shown in Table 2, the recovery was in the range of 92% to 103% with a relative standard deviation below 2.4%. The results indicate that the probe and the proposed detection method are applicable for glucose detection in real samples.

## 4. Conclusions

This study presents a simple, inexpensive, and portable sensor for measuring Clz by modifying the working electrode of a commercial glucose test strip. The CTS was modified by removing the enzyme-based biorecognition layer and then applying a drop-cast of MWCNT onto the surface of the working electrode along with Nafion. The sensor’s electrochemical properties and performance were evaluated using CV, DPV, SWV, and amperometric measurements. The linear detection range between 0.1 and 5 μM highlights the sensor’s potential for practical use in diagnostics. Additionally, the sensor showed long-term stability and satisfactory recoveries in spiked human blood serum. This research demonstrates a simple fabrication method for creating CTS electrodes for the detection of drugs and other analytes, which could pave the way for the development of sensors for drug monitoring.

## Figures and Tables

**Table 1:** Comparison of the performance characteristics of various electrochemical methods for Clz sensors with proposed electrodes.

Electrode	Technique	LR * (µM)	LD * (nM)	Ref.
Nf/MWCNT on CTS	CV DPV SWV i-t	0.1–5.0 0.1–5.0 0.1–5.0 0.1–5.0	116 104 83 192	This work
RDTNP *	SWV	0.9–40	43	[9]
PPFE *	DPV	50–500	6000	[32]
ISE *	Potentiometry	10–10,000	3400	[33]
TiO_2_/CPE *	DPV	0.5–45	61	[34]
PGE *	DPV	0.0095–1.5	2.86	[35]
RuTiO_2_/CPE *	SWV	09–40	0.43	[31]

* LR: linear range, Ref: references, ISE: ion-selective electrode, TiO_2_/CPE: titanium oxide modified carbon paste electrode, SWV: square-wave voltammetry, PPFE: polypyrrole fiber electrode, RDTNP: ruthenium doped titanium nanoparticle.

**Table 2:** Determination of Clz in spiked serum samples (1 μM Clz in 10 mM PBS pH 7.4).

Spiked (µM)	Detected (µM)	Recovery (%)	RSD * (%) n:3
0	0	Not detected	-
0.25	0.23	92	2.4
0.5	0.47	94	2.1
1	1.03	103	1.9

* Relative standard deviation.

## Data Availability

All data will be available and shared upon request.
